# RNA-binding proteins potentially regulate the alternative splicing of apoptotic genes during knee osteoarthritis progression

**DOI:** 10.1186/s12864-024-10181-w

**Published:** 2024-03-19

**Authors:** Zheng Zhang, Limei Dong, Hai Tao, Yusong Dong, Wei Xiang, Fenghua Tao, Yingchun Zhao

**Affiliations:** 1 Department of Orthopedics, Renmin Hospital of Wuhan University, 238, Jiefang Road, Wuchang District, 430060 Wuhan, Hubei China; 2 School of Basic Medical Sciences, Wuhan University, 430071 Wuhan, Hubei China

**Keywords:** Knee osteoarthritis, RNA binding protein, Alternative splicing, Transcriptome, Differentially expressed genes, Apoptosis, Covariation

## Abstract

**Background:**

Alternative splicing (AS) is a principal mode of genetic regulation and one of the most widely used mechanisms to generate structurally and functionally distinct mRNA and protein variants. Dysregulation of AS may result in aberrant transcription and protein products, leading to the emergence of human diseases. Although considered important for regulating gene expression, genome-wide AS dysregulation, underlying mechanisms, and clinical relevance in knee osteoarthritis (OA) remain unelucidated. Therefore, in this study, we elucidated and validated AS events and their regulatory mechanisms during OA progression.

**Results:**

In this study, we identified differentially expressed genes between human OA and healthy meniscus samples. Among them, the OA-associated genes were primarily enriched in biological pathways such as extracellular matrix organization and ossification. The predominant OA-associated regulated AS (RAS) events were found to be involved in apoptosis during OA development. The expression of the apoptosis-related gene BCL2L13, XAF1, and NF2 were significantly different between OA and healthy meniscus samples. The construction of a covariation network of RNA-binding proteins (RBPs) and RAS genes revealed that differentially expressed RBP genes LAMA2 and CUL4B may regulate the apoptotic genes XAF1 and BCL2L13 to undergo AS events during OA progression. Finally, RT-qPCR revealed that CUL4B expression was significantly higher in OA meniscus samples than in normal controls and that the AS ratio of XAF1 was significantly different between control and OA samples; these findings were consistent with their expected expression and regulatory relationships.

**Conclusions:**

Differentially expressed RBPs may regulate the AS of apoptotic genes during knee OA progression. XAF1 and its regulator, CUL4B, may serve as novel biomarkers and potential therapeutic targets for this disease.

**Supplementary Information:**

The online version contains supplementary material available at 10.1186/s12864-024-10181-w.

## Background

As a common degenerative disorder, osteoarthritis (OA) encompasses several physiological and biomechanical variations in the joint, including articular reconstruction, chondral deterioration, and formative hyperostosis, resulting in symptomatic manifestations [1]. Although it incurs substantial social and individual economic costs, OA is often overlooked. OA does not occupy an essential position in global prevention strategies for noninfectious diseases; however, it often coexists with cardiovascular diseases and neurological and psychological disorders, worsening the morbidity and progression of these diseases [2]. As the most common arthritis type, OA generally results in joint pain and provokes severe functional disabilities owing to eroded cartilage and meniscus tissues. However, the pathogenic mechanism of underlying OA has not been comprehensively elucidated and disease-modifying therapies remain unknown. Arthroplasty is the only effective therapeutic modality for end-stage OA [3]. Recently, a study revealed that the antagonizing nerve growth factor and its receptor tropomyosin-related kinase A can be effectively used to relieve OA-induced knee joint pain [4]. In a meta-analysis covering 6860 clinical patients, Schmitz et al. summarized the findings of 183 studies on using stem cells to differentiate them into osteochondrocytes for treating knee OA [5]. Nevertheless, no treatment modality significantly alters disease progression or effectively prevents arthroplasty in end-stage OA. Subsequently, novel biomarkers or therapeutic targets should be further discovered in present basic research and for treating OA in clinical settings.

Alternative splicing (AS) refers to a cellular process in which the exons of primary transcripts are differentially spliced to generate structurally and functionally distinct mRNA and protein variants. AS is a principal mode of genetic regulation and possibly one of the most widely used mechanisms; it accounts for the complexity of large molecules and cells in higher eukaryotes [6, 7]. AS produces various transcripts from approximately 95% of multiexon human genes, varying the transmission of genetic information [8]. Dysregulation may result in aberrant transcription and protein products; these products have been implicated in several human diseases, including Parkinson’s disease, Lou Gehrig’s disease, premature ageing, and carcinoma [9–11]. BRCA1 (breast cancer 1, early onset), CFTR (cystic fibrosis transmembrane conductance regulator), HPRT1 (hypoxanthine phosphoribosyl transferase 1), MAPT (microtubule-associated protein tau), SMN1 (survival of motor neuron 1) etc. are the known human disease-associated genes identified as biomarkers that undergo exonic splicing [12].

In the nucleus, some RNA-binding proteins (RBP) often function as AS regulators in a particular way in the disease course [13, 14]. Functional disruptions in RBPs may be a major cause or consequence of a disease [15, 16]. CUGBP Elav-like family (CELF) and muscleblind-like (MBNL) proteins are some examples of RBPs functioning as splicing regulators; they play vital roles in myotonic dystrophy by promoting opposite effects on the splice site or exon usage, affecting mRNA localization and stability in the cytoplasm [17]. Therefore, understanding the underlying roles of RBPs during RNA processing is vital to elucidate the mechanisms underlying the occurrence and outcomes of human diseases [18, 19]. It has been reported that RBPs regulate mRNA splicing in cartilage tissues of OA patients, potentially affecting cell adhesion and chondrocyte matrix degradation [20, 21]. These findings highlighted the important role of gene splicing in OA progression, however, the splicing pattern and its regulatory mechanism during articular degeneration in knee OA remain unelucidated, possibly having important guiding significance and research value for disease diagnosis, treatment, and prognosis.

Based on the background, in the present study, we hypothesized that post-transcriptional gene regulation in knee OA affects morphological and pathological changes in the cartilage. Several differentially expressed RBPs (DERBPs) present in knee OA may regulate the AS of disease-associated genes, leading to the distinct expression of proteins and playing roles in disease progression. To reveal the mystery of transcriptome dysregulation in knee OA, we analyzed a series of knee OA-related data to examine the transcriptome of degenerative joints in humans via a data-based assembly of sequencing reads. Analysis revealed the differentially expressed genes (DEGs) and biological pathways enriched between patients with knee OA and healthy individuals. By identifying the predominant regulated AS (RAS) event, we discovered the RAS genes and their biological pathways in knee OA. Furthermore, we investigated AS dysregulation by DERBPs, constructed a covariation network of RBPs and RAS genes in knee OA, and validated gene expression and splicing via RT-qPCR by using the menisci of patients with knee OA and healthy individuals. Our study findings provide a significant resource for exploring AS dysregulation in knee OA and the possible therapeutic targets for treating early-stage OA.

## Results

### RNA sequencing (RNA-seq) of human meniscus cells in OA and healthy samples

**Fig. 1:**
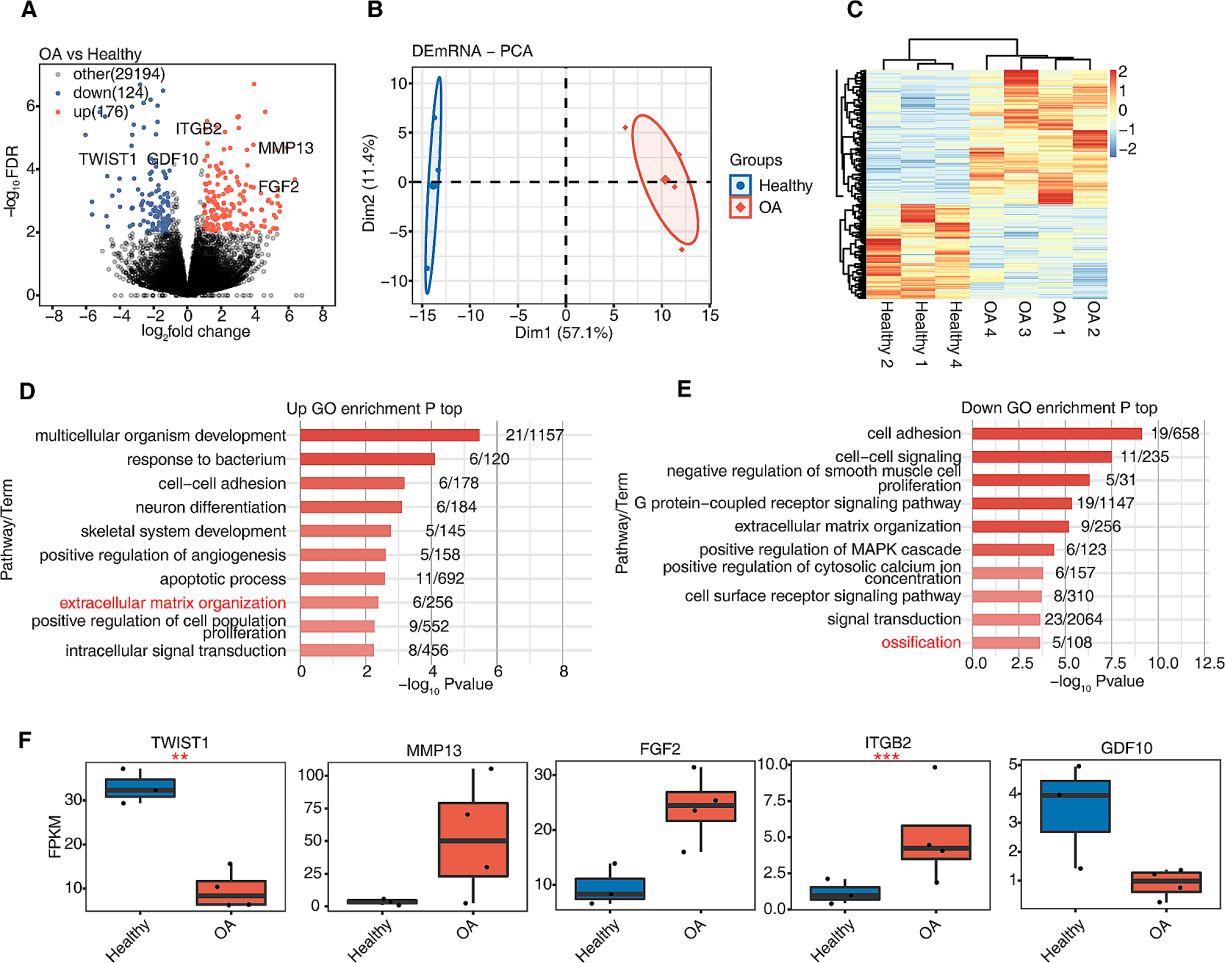
RNA sequencing of human meniscus cells in osteoarthritis (OA) and healthy samples. (A) Volcano plots presenting all differential expressed genes (DEGs) between OA and healthy samples. false discovery rate (FDR) ≤ 0.01 and fold change ≥ 2 or ≤ 0.5. (B) Principal component analysis (PCA) based on fragments per kilobase of exon per million mapped fragments (FPKM) value of all DEGs. The ellipse for each group is the confidence ellipse. (C) Hierarchical clustering heatmap showing the expression of all DEGs. (D) Bar plot showing the most enriched GO pathways of the upregulated genes. (E) Bar plot showing the most enriched GO pathways of the downregulated genes. (F) Boxplot showing FPKM of 5 DEGs. ^*^: *p* < 0.05, ^**^: *p* < 0.01, ^***^: *p* < 0.001

To elucidate the functional role of AS regulatory mechanism in OA, the RNA-seq data of four human OA and four healthy meniscus samples were downloaded and analyzed. Considering the presence of a serious outlier in healthy samples, we eliminated this sample and performed transcriptome analysis of DEGs using four OA samples and three healthy samples based on their gene expression. Three hundred DEGs were identified between OA and healthy samples; 176 genes were upregulated and 124 genes were downregulated. Among the upregulated genes, MMP13, FGF2, and ITGB2 were associated with OA. Furthermore, among the downregulated genes, TWIST1 and GDF10 were associated with OA (Fig. 1A). Based on the FPKM value of all DEGs, PCA revealed a clear distinction between the OA and healthy groups (Fig. 1B). Furthermore, hierarchical clustering of the expression of all DEGs via heatmap revealed significant differences between OA and healthy samples (Fig. 1C). Therefore, our results suggest that differential gene expression reflects heterogeneity across OA and healthy control samples.

To clarify the biological roles of these DEGs in OA development, the dysregulated genes were extracted to perform functional enrichment analysis (Fig. 1D-E; Supplemental Fig. S1A-B). Upregulated genes were mostly enriched in signaling pathways, including apoptosis, development of multicellular organisms, response to bacterium, extracellular matrix organization, cell–cell adhesion, neuronal differentiation, development of the skeletal system, positive regulation of angiogenesis, positive regulation of cell proliferation, and intracellular signal transduction. Among them, the OA-associated genes MMP13, FGF2, and ITGB2 were primarily enriched in extracellular matrix organization (Fig. 1D). On the other hand, the downregulated genes were mostly enriched in signaling pathways such as cell adhesion, cell–cell signaling, extracellular matrix organization, negative regulation of smooth muscle cell proliferation, the G protein-coupled receptor signaling pathway, positive regulation of the MAPK cascade, the cell surface receptor signaling pathway, positive regulation of cytosolic calcium ion concentration, signal transduction, and ossification. The OA-associated DEGs, including TWIST1 and GDF10, were primarily enriched in ossification (Fig. 1E). Figure 1F illustrates the expression of five OA-associated genes based on FPKM. The expression of the downregulated gene TWIST1 and the upregulated gene ITGB2 exhibited significant differentiation (*p* < 0.01). Therefore, DEGs may affect OA development by participating in extracellular matrix organization and ossification.

### Identification of the predominant RAS events between OA and healthy samples

**Fig. 2:**
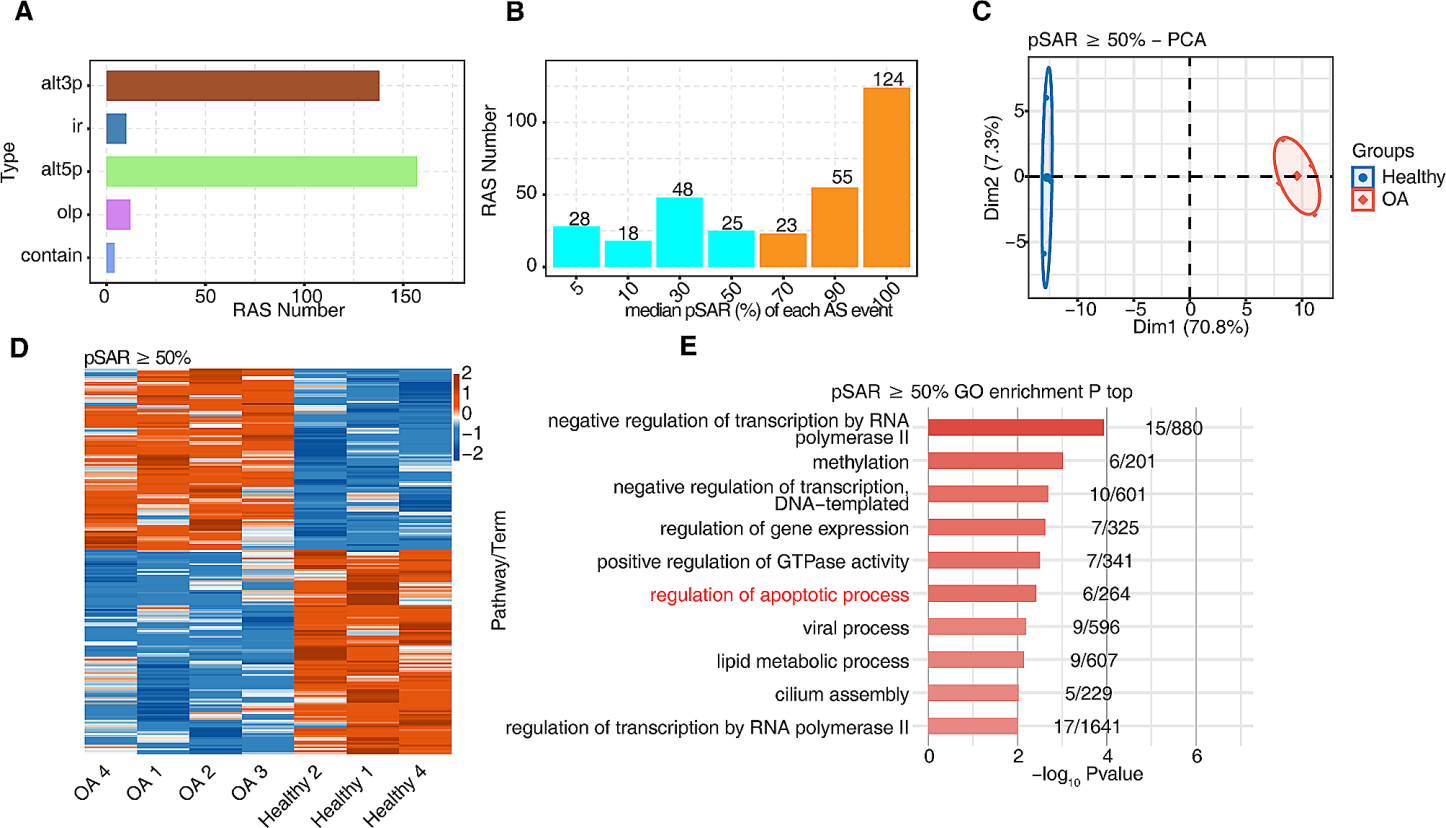
Identification of predominant regulatory alternative splicing (RAS) events between OA and healthy samples. (A) Bar plot showing the number of different RAS types identified by SUVA in OA samples. (B) Bar plot showing RAS with different pSAR (proportion of each SUVA AS event reads). RAS with pSAR ≥ 50% were labeled. (C) Principal component analysis (PCA) based on RAS with pSAR ≥ 50%. The ellipse for each group is the confidence ellipse. (D) The heatmap showing the splicing ratio of RAS of all samples (pSAR ≥ 50%). (E) Bar plot exhibiting the most enriched GO pathways of the RAS with pSAR ≥ 50%.

SUVA, a recently introduced AS analysis software, was used to identify and analyze the different AS events between OA and healthy samples. Figure 2A and Supplemental Fig. S2A illustrate the several different RAS types identified in the transcripts in OA and healthy samples, respectively. alt3p and alt5p were the primary RAS events in the present study. Effectively, a splicing event involves two transcripts that may occupy a small fraction of the overall gene expression. We tried to identify more predominant transcripts that underwent splicing with a higher pSAR via SUVA. Figure 2B demonstrates the RAS numbers accounting for the different proportions of all reads, and thereinto, 202 predominant OA-associated RAS events with pSAR ≥ 50% were screened for subsequent analysis. Based on the selected RAS, the splicing ratio of OA-associated RAS events was used to perform PCA to clearly differentiate the OA and healthy samples. PCA showed that splicing in OA samples was significantly different compared with that in healthy samples, revealing that OA-associated RAS may be responsible for disease response (Fig. 2C). Figure 2D demonstrates a heatmap displaying the splicing ratio of all RAS events with pSAR ≥ 50% of all samples. To determine the implied function of these RAS, we selected the genes that underwent AS for functional enrichment analysis. Negative regulation of transcription by RNA polymerase II, methylation, regulation of gene expression, positive regulation of GTPase activity, negative regulation of transcription, regulation of apoptotic process, viral process, lipid metabolic process, DNA-templated, cilium assembly, and regulation of transcription by RNA polymerase II were identified as the most enriched GO biological pathways. We observed that apoptosis may be relevant to OA occurrence (Fig. 2E). Supplemental Fig. S2B displayed the top 10 enriched KEGG pathways of RAS (pSAR ≥ 50%).

### AS of apoptotic genes in osteoarthritis

**Fig. 3:**
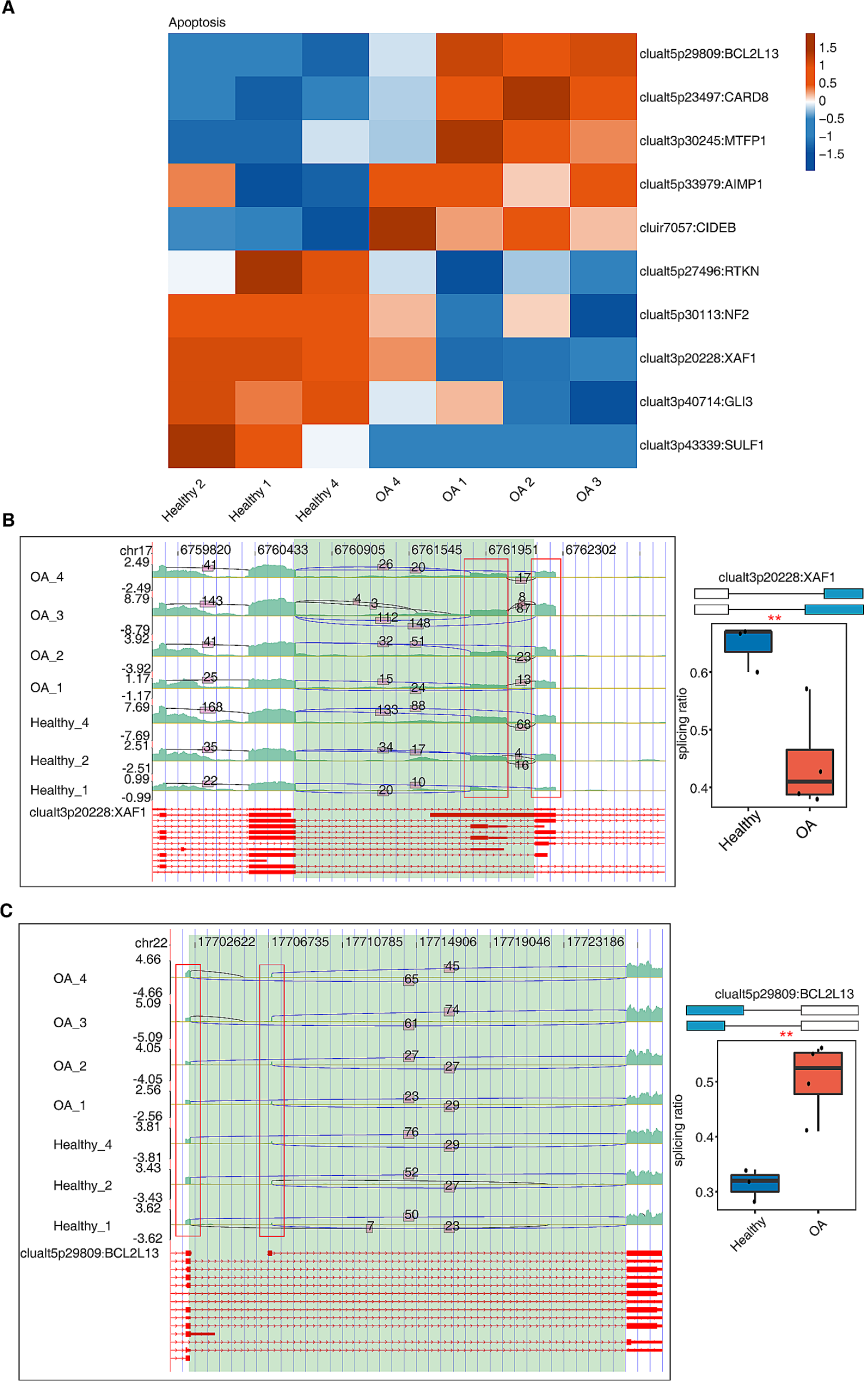
Alternative splicing (AS) of apoptotic genes in osteoarthritis. (A) The heatmap showing splicing ratio of RAS events of all samples in apoptotic pathway. (B-C) The read distribution and splicing ratio of apoptotic genes XAF1, BCL2L13. The red boxes in the left panel indicate the splicing area of the difference; the boxplots in the right panel show the splicing ratio. ^*^p < 0.05, ^**^p < 0.01

Based on the OA-related biological pathways that were screened, the RAS events in apoptosis were investigated. The heatmap of the splicing ratio of the RAS of all samples in the apoptotic pathway revealed that XAF1, BCL2L13, and NF2 had significantly differential expression among the genes that underwent AS (Fig. 3A). Figure 3B-C and Supplemental Fig. S3 illustrate the read distribution and splicing ratio of these three apoptotic genes. The results suggest that the occurrence of apoptosis-associated AS events was significantly different between healthy and OA samples (all *p* < 0.05). The expression of the apoptosis-related gene BCL2L13 was upregulated in OA samples than in healthy samples, whereas the expressions of the other two apoptotic genes, namely, XAF1 and NF2, were relatively downregulated.

### Construction of a covariation network of RBPs and RAS in osteoarthritis

Next, to investigate the possible AS dysregulation in knee OA, we identified the DERBPs in OA and constructed a covariation network of RBPs and RAS based on RBP gene expression and the splicing ratio of RAS genes.

**Fig. 4:**
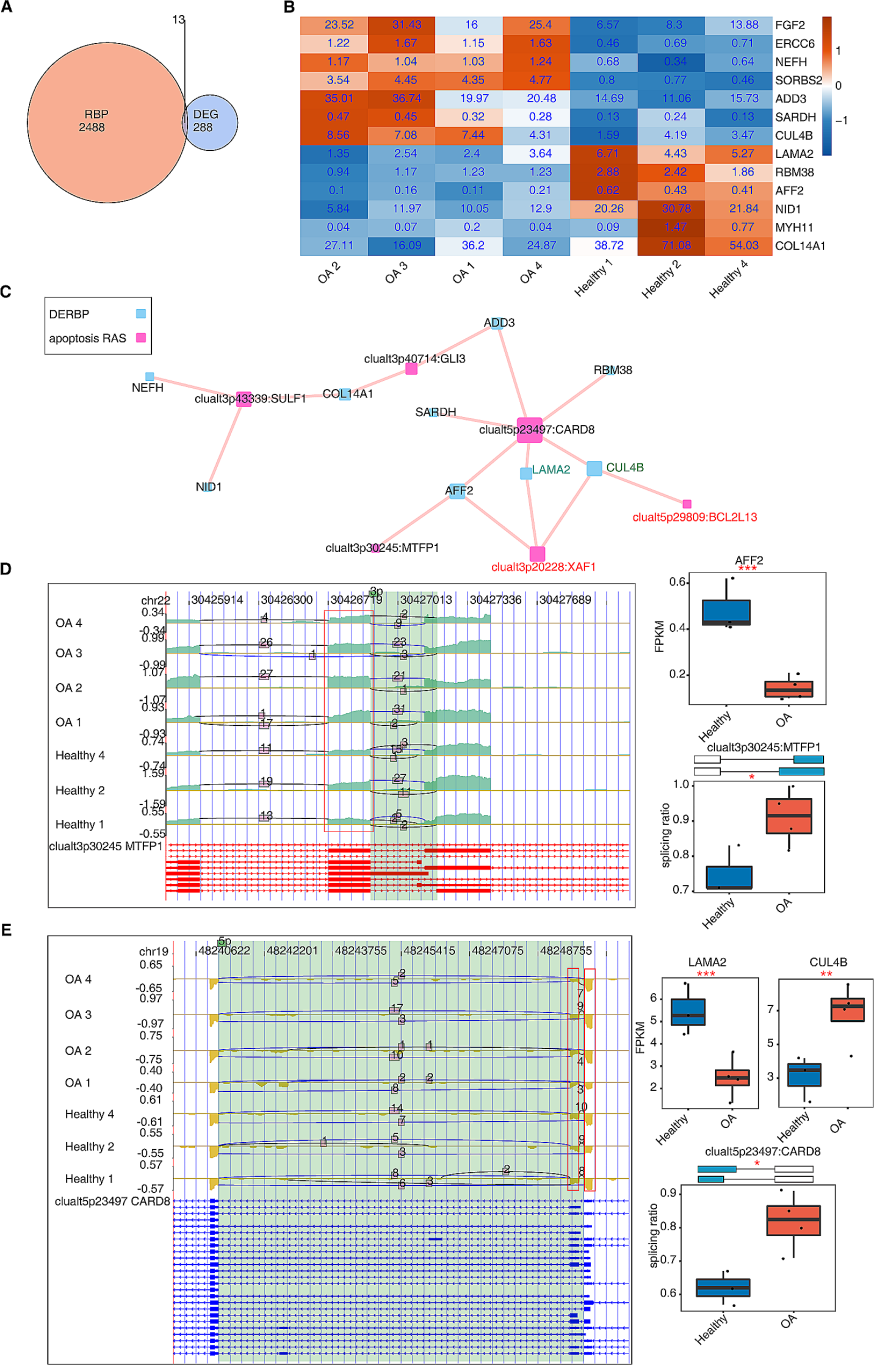
Covariation analysis of differentially expressed RNA binding protein (DERBP) and regulatory alternative splicing (RAS) in apoptotic pathway of OA. (A) Venn diagram showing the overlap of RBP and DEG. (B) Heatmap showing the expression profile of DERBP in apoptotic pathway of osteoarthritis. (C) Covariation network of DERBPs and apoptosis-associated RAS (pSAR ≥ 50%). Cutoffs of *p* ≤ 0.01 and Pearson coefficient ≥ 0.6 ≤ -0.6 were applied to identify the covariation pairs. (D) The read distribution of the apoptotic RAS gene MTFP1 is showing in the left panel. The red box indicates the splicing area of the difference. The boxplots in the right panel show the FPKM of the coexpressed RBP gene AFF2 and the splicing ratio of MTFP1. ^*^: *p* < 0.05, ^**^: *p* < 0.01, ^***^: *p* < 0.001. (E) The reads distribution of the apoptotic RAS gene CARD8 is showing in the left panel. The red boxes indicate the splicing area of the difference. The boxplots in the right panel show the FPKM of the coexpressed RBP genes LAMA2 and CUL4B and the splicing ratio of CARD8. ^*^: *p* < 0.05, ^**^: *p* < 0.01, ^***^: *p* < 0.001

The 2501 human RBP genes and DEGs identified in our study were overlapped, resulting in the identification of 13 DERBP genes (Fig. 4A). Figure 4B illustrates that among the 13 RBP genes, the expression changes in CUL4B, NID1, LAMA2, AFF2, COL14A1, and NEFH were higher between OA and healthy samples compared with the other corresponding genes in the heatmap. To further explore the possible regulatory relationships in the apoptotic pathway, we constructed a covariation network of DERBPs and apoptosis-associated RAS (Fig. 4C). Using this network, we observed that the RBP gene LAMA2 may significantly regulate the apoptotic gene XAF1 to undergo AS events; on the other hand, the RBP gene CUL4B may significantly regulate both XAF1 and BCL2L13, apoptotic genes, to undergo AS events during OA progression. Pearson’s correlation analysis identified LAMA2–XAF1 (correlation coefficient = 0.93, *p* < 0.01), CUL4B–XAF1 (correlation coefficient = 0.88, *p* < 0.01), and CUL4B–BCL2L13 (correlation coefficient = 0.85, *p* < 0.01) as the covariation RBP–AS relationship pairs in this network. Figure 4D-E and Supplemental Fig. S4 display the read distribution and splicing ratio of other three apoptotic RAS genes: MTFP1, CARD8, and SULF1, respectively. These genes displayed smaller AS read number but significant covariation with the corresponding RBP genes. As a result, MTFP1 and AFF2 were significantly coexpressed, CARD8 was coexpressed with LAMA2 and CUL4B, and SULF1 was coexpressed with NEFH, NID1, and COL14A1 (all *p* < 0.05). Furthermore, we observed that the expressions of the RBP genes CUL4B and NEFH and apoptotic RAS genes MTFP1 and CARD8 were upregulated in OA samples compared with that in healthy samples. However, the expressions of the RBP genes AFF2, LAMA2, NID1, and COL14A1 and the apoptotic RAS gene SULF1 were relatively downregulated.

### Validation of the expression of RBP and RAS genes

Finally, meniscus samples were collected from three patients with OA (2 men and 1 woman, mean age: 75.2 ± 6.8 years) who underwent TKA and three young patients (2 men and 1 woman, mean age: 23.2 ± 4.1 years) with sports injuries who underwent arthroscopic partial meniscectomy as normal controls for RT-qPCR. Table 1 presents the primers for qPCR analysis.

**Table 1:** RT-qPCR primers for expression of RBP and RAS genes

Gene	Primer	Sequence (5ʹ-3ʹ)
GAPDH	Forward	GGTCGGAGTCAACGGATTTG
GAPDH	Reverse	GGAAGATGGTGATGGGATTTC
LAMA2	Forward	CACTGCTGTCTATGATGCT
LAMA2	Reverse	ATGTAGATGCTGGGTTTGG
CUL4B	Forward	TATTCCAGTTGCCTGCTTG
CUL4B	Reverse	AAAGGAACTCCAGGTCTCT
XAF1-M/AS	Forward	GCCCTGTTGAGTGTAAGTTCTGC
XAF1-M	Reverse	AGGACTGGTTTCTTTCCCGAG
XAF1-AS	Reverse	ATTCTTTCCCCTTTCCCGAG
BCL2L13-M	Forward	AGGTGGCTGGGGCACTGTGT
BCL2L13-AS	Forward	AGTCCACTGTGGGCACTGTGT
BCL2L13-M/AS	Reverse	CTGGGCAGGATGTAAATGTC

RBP: RNA binding protein; RAS: regulatory alternative splicing; GAPDH: glyceraldehyde-3-phosphate dehydrogenase (internal reference); M: model splicing; AS: altered splicing

**Fig. 5:**
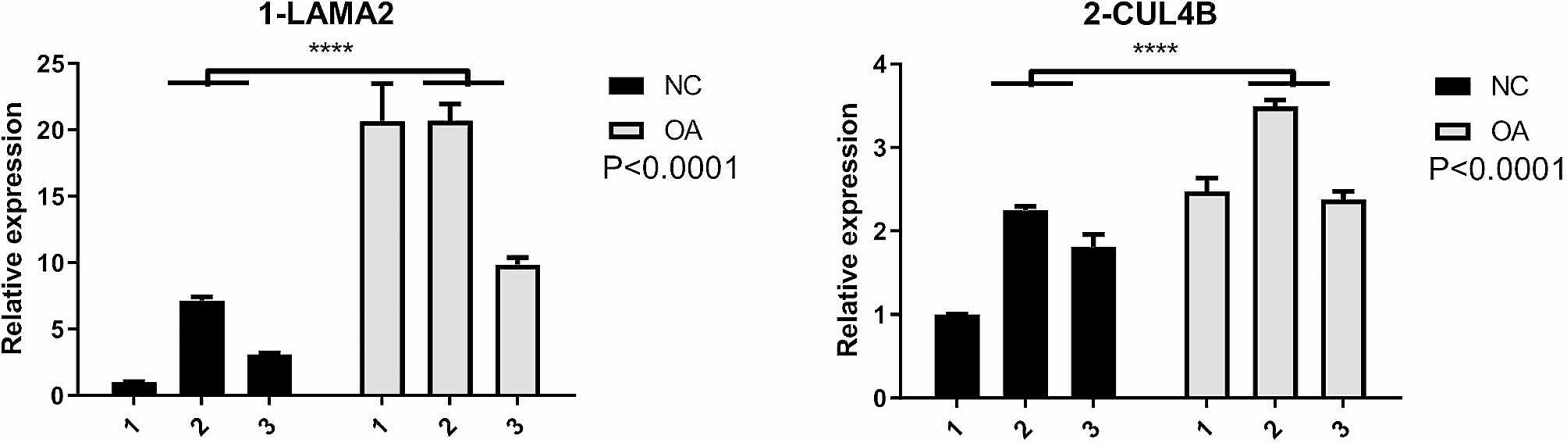
Expression of RBP genes LAMA2 and CUL4B in normal control (black bars) and OA meniscus samples (gray bars); NC: normal control; OA: osteoarthritis; ^*^: *p* < 0.05, ^**^: *p* < 0.01, ^***^: *p* < 0.001, ^****^: *p* < 0.0001

The RBP genes LAMA2 and CUL4B were both differentially expressed between control and OA samples. Compared with the control samples, LAMA2 expression was increased in OA samples; this was contradictory to the expected result of LAMA2 as a downregulated gene (Fig. 5, *p* < 0.0001). However, the expression of CUL4B, a predicted upregulated gene, was increased in OA samples, consistent with the expected result (Fig. 5, *p* < 0.0001).

**Fig. 6:**
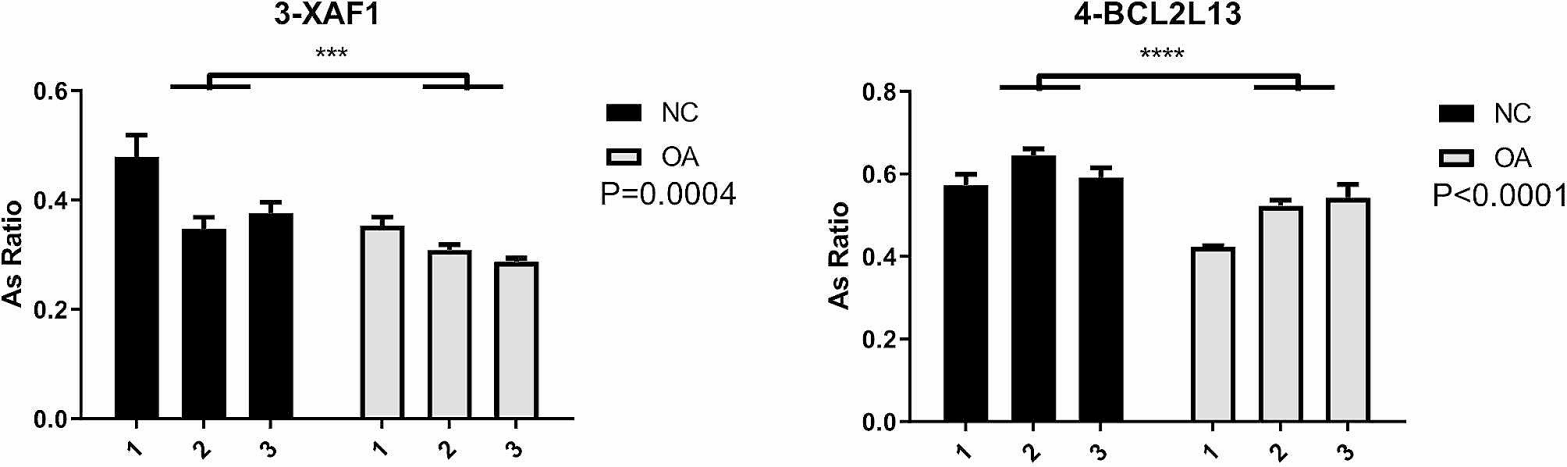
AS ratio of RAS genes XAF1 and BCL2L13 in normal control (black bars) and OA meniscus samples (gray bars); NC: normal control; OA: osteoarthritis; ^*^: *p* < 0.05, ^**^: *p* < 0.01, ^***^: *p* < 0.001, ^****^: *p* < 0.0001

The AS ratio of the RAS gene XAF1 was significantly different between control and OA samples, consistent with its expected downregulation in OA samples (Fig. 6, *p* = 0.0004). Furthermore, the AS ratio of BCL2L13 was significantly different between control and OA samples; however, its expression was downregulated in OA samples, contradictory to its expected upregulation (Fig. 6, *p* < 0.0001).

## Discussion

Since AS and its regulator, RBPs, were identified to play vital roles in the development of human diseases, increasing studies have focused on the application of sequencing technology to elucidate the relationship between the post-transcriptional regulation of genes and human diseases. However, owing to the limited application and development of traditional sequencing techniques, no further cognitive breakthroughs have been achieved in the AS of human genes. Increasing research on post-transcriptional regulation and the extensive application of new-generation sequencing techniques have revealed that AS modulation is substantially more complicated than expected, particularly when considering diseases such as cancers and degenerative diseases [22–25]. OA, also called hypertrophic or senile arthritis, is a type of degenerative disease caused by aging, obesity, strain, trauma, congenital joint abnormalities, articular deformity, and many other factors; this disrupts the articular cartilage and meniscus and causes reactive hyperplasia of the joint edge and subchondral bone [4]. This type of slow-progressing degeneration may eventually lead to the irreversible degradation of periarticular structures, including bony and soft tissues. Although there are many studies on OA pathogenesis, treatment, and prognosis, the roles of AS and its regulatory mechanisms in knee OA development remain uninvestigated.

In the present study, we analyzed the RNA-seq data of human meniscus samples from patients with knee OA and healthy individuals. OA-associated DEGs were observed to be significantly enriched in the signaling pathways related to extracellular matrix organization and ossification. Meanwhile, OA-associated RAS genes participate in apoptosis during disease development. To further elucidate the regulation of OA-associated AS, we identified the DERBP genes in OA and then constructed a covariation network of RBPs and RAS. The covariation RBP–AS relationship pairs revealed that RBP genes may regulate apoptotic genes to undergo AS events during transcription; this, in turn, affects OA progression. The validated RAS gene XAF1 and its regulator, the RBP gene CUL4B, may be novel biomarkers and potential therapeutic targets for this disease. Our study results provide the first portrait of aberrant AS events in OA meniscus samples and the covariation network of RBPs and RAS in OA. These outcomes provide novel insights into OA pathogenesis, diagnosis, and therapy.

In fact, changes in the extracellular matrix are associated with early OA. Brophy et al. used RNA microarrays to identify the differentially expressed transcripts in OA and non-OA meniscus samples [26]. Brophy et al. reported that vascular endothelial growth factor A (VEGFA) is the most highly repressed transcript gene in the OA meniscus, serving as an angiogenesis marker [27]. In patients with knee OA, VEGFA signaling is attenuated, and the extracellular matrix is lost; this suggests that the extracellular matrix acts as a key point in vascular structuring and tissue repair owing to changes in the morphology and composition of extracellular matrix macromolecules, indicating the early onset of OA. In addition, previous animal experiments revealed the presence of changes in cellular distribution and extracellular matrix deposition in the medial meniscus of rabbit OA models in the early disease stage [28]. In the present study, ITGB2, as an upregulated gene, was noted to be enriched in the biological pathways associated with extracellular matrix organization. Furthermore, a similar study has confirmed that ITGB2 may be closely associated with OA pathogenesis and a possible therapeutic target [29]. Besides, the signaling pathway associated with ossification plays a vital role in OA development. Aggravated ossification of the meniscus may alter the pressure of the subchondral bone in Hartley guinea pig OA models and worsen the destruction of the joint compartment to some extent [30, 31]. In our study, the downregulated gene TWIST1 was noted to be enriched in the ossification pathway, provoking the disruption of joint function. Notably, previous studies have revealed that as a transcription factor, TWIST1 is dysregulated during osteoblast differentiation, altering intrinsic osteoblast behavior and OA progression [32]. Based on the regulatory effect on TWIST1 expression, piperlongumine, a beneficial and effective treatment, has been proposed as a new approach for OA therapy [33]. Therefore, structural and metabolic changes in the extracellular matrix and meniscus ossification may serve as new diagnostic biomarkers for early OA, providing a novel thought for targeted treatment.

Apoptosis, a biological process in which the RAS genes were most frequently involved in the present study, plays an essential role during OA progression. A previous study revealed an increased incidence of apoptosis in the osteoarthritic cartilage [34]. Apoptosis-induced morphological changes have been observed in the chondrocytes in degenerative cartilage, suggesting a pathway for OA pathology [35]. Our findings are consistent with those of another study that revealed that BCL2L13, a proapoptotic member of the BCL-2 family of apoptosis protein, plays a role in the mitochondrial apoptosis pathway, which is altered in OA [36]. By upregulating BCL2L13 expression, the long noncoding RNA SNHG15 can inhibit OA progression [37]. Subsequently, BCL2L13 variants can, by extension, affect OA susceptibility and progression. On the other hand, the XAF1 gene exerts its effects as an OA-associated apoptotic assistor, suggesting that it facilitates p53-mediated cell apoptosis by improving post-translational modifications [38]. Furthermore, in the present study, we observed that RAS genes were simultaneously enriched in the negative regulation of the biological pathways associated with OA progression. Malemud reported the negative regulation of the JAK/STAT signaling pathway affected the apoptosis of activated immune and synovial cells during abnormal survival; he hypothesized that the increased apoptosis of chondrocytes is associated with OA [39]. Based on this, we summarize that the biological processes of apoptosis and negative regulation may jointly act on OA progression and evolve into potential therapeutic targets in the future.

The construction of a covariation network of RBPs and RAS in this study, suggests that the dysregulation of RBP expression affects the AS of relevant downstream genes. The identified RBP–AS pairs, namely, LAMA2–XAF1, CUL4B–XAF1, and CUL4B–BCL2L13, should be considered biomarkers for targeted therapy for OA. The mutation of LAMA2, a gene encoding laminin-α2, can lead to congenital muscular dystrophy [40, 41]. Although the expression of the apoptotic gene LAMA2 was upregulated in OA meniscus samples in our study, which was contradictory to the expected results, its role in OA should be further investigated. Mi et al. reported that CUL4B upregulates RUNX2, a master transcription factor, to promote the osteogenic differentiation of human periodontal ligament stem cells by epigenetically repressing miR320 and miR-372/373-3p expression [42]. Furthermore, Wang et al. revealed that Huangqin Qingre Chubi capsule, a traditional Chinese medicine compound, interferes with the effects of circ_0015756 on rheumatoid arthritis (RA) pathogenesis by inhibiting the specific target CUL4B; this suggests its good therapeutic effect on RA [43]. However, for the identified biomarkers in OA, such as CUL4B and XAF1, there are no documented reports of their covariable expression in other disease. This specificity is crucial for their application in OA-specific diagnosis and treatment. By constructing a covariation network and the expression of related genes in meniscus samples, we assume that the validated RBP gene CUL4B can induce AS events during transcription by regulating the apoptotic gene XAF1, thereby resulting in OA occurrence or development. Our findings also provide new ideas for specific genetic diagnosis and targeted treatment of OA. For example, utilizing gene probe technology as a diagnostic tool to detect the expression of OA-related covariable RBP-AS gene pairs in patients would be a new option for screening or early diagnosis of OA. Meanwhile, developing specific drugs that selectively affect the expression of OA-related RBP genes or interfere with their regulation of AS genes to inhibit chondrocyte apoptosis may slow down or even block the disease process, thus allowing for targeted therapy.

This study has several limitations. Owing to the absence of publicly available sequencing data and the difficulty in obtaining healthy meniscus tissues, the research relied on a constrained sample size. We were unable to use more additional independent cohorts to verify the generalizability of the experimental findings, while also being unable to take into account potential confounding factors such as age, sex, OA severity, comorbid conditions, environmental and lifestyle influences. Future study should identify and validate more RBPs and their influence on AS to enhance the extensibility and robustness of the findings, while establishing mixed-effect models to explore the effect of confounding factors on gene expression and AS events when sufficient samples are obtained. Furthermore, this study concentrated solely on apoptosis caused by altered splicing patterns, lacking the exploration of other potential biological pathways implicated in the pathogenesis of OA as well as in vitro or in vivo functional experiments. Future research could address these limitations by establishing animal models, culturing more human meniscal chondrocytes, and validating more target genes and biological pathways to conclusively demonstrate the effects of the identified AS events and RBP regulations on the pathogenesis of OA. In addition, the cross-sectional design in the present study restrained our exploration of the role that the regulation of RBPs on AS plays in the overall development of OA disease. Future longitudinal or cohort-sequential designs are expected to track changes in AS events and RBP expression throughout OA progression.

## Conclusions

In this study, we identified OA-associated DEGs and analyzed the aberrant AS events in OA meniscus samples for the first time based on RNA-seq data. Significant differences in RAS between OA and healthy samples suggest the regulatory effect of the splicing machinery in OA. In particular, the first reported covariation network of RBP–RAS in meniscus samples revealed that DERBPs may regulate the AS of apoptosis-associated genes during knee OA progression, providing novel insights into the development of gene-targeted diagnostic and therapeutic strategies for osteoarthritic diseases.

## Methods

### Public data retrieval and processing

The publicly available sequence data files of four human OA meniscus samples and four healthy meniscus samples from GSE185064 were downloaded from the Sequence Read Archive (SRA) (https://www.ncbi.nlm.nih.gov/geo/query/acc.cgi?acc=GSE185064) [44]. SRA Run files were converted to fastq format using the NCBI SRA Tool fastq-dump. Raw reads were trimmed to low-quality bases using the FASTX-Toolkit (v.0.0.13; http://hannonlab.cshl.edu/fastx_toolkit/). Then, clean reads were assessed using FastQC (http://www.bioinformatics.babraham.ac.uk/projects/fastqc/). Clean reads were aligned to the human genome using HISAT2 [45]. Uniquely mapped reads were used to calculate the read number and fragments per kilobase of exon per million mapped fragments (FPKM), which reflect gene expression.

### Identification of DERBPs between OA and healthy samples

DEseq2 software was used to screen the raw count data of DEGs; this software can analyze differential gene expression. Based on fold change ≥ 2 or ≤ 0.5 and false discovery rate (FDR) ≤ 0.01, the DEGs were identified. Then, based on a catalog of RBPs retrieved from four previous studies, the expression profile of DERBPs was screened from all DEGs [46–49].

### AS analysis

Based on the changes in splicing site usage, five different AS event models, namely, alt3p, alt5p, olp, contain, and ir patterns, were defined and quantified using a previously described splice site usage variation analysis (SUVA) pipeline [50]. “alt3p” means that the model has an alternative 3ʹ splice site and a shared 5ʹ splice site. Similarly, “alt5p” means that the model has an alternative 5ʹ splice site and a shared 3ʹ splice site. “olp” refers to a model with two distinct splice sites; however, a part of these sites are overlapped. “contain” is a model with two different splice sites; however, one site is present within the other one. “ir” refers to a model in which both splice sites can be used or not used, similar to the “intron retention” event in classical definition. We used the SUVA pipeline to detect AS events from short read RNA-seq data based on the five different splicing patterns described above. In addition to directly quantifying simple AS events, this pipeline was also able to decompose the complex AS profiles into the five types of splice junction pairs and re-quantify each sub-splicing events. A previously described method based on corresponding algorithms was used to calculate and quantify the proportion of each SUVA AS event read (pSAR) as well as the splicing ratio of the target genes [50].

### Covariation analysis

All DERBPs and RAS genes were subjected to covariation analysis (pSAR ≥ 50%). Meanwhile, the Pearson’s correlation coefficient between DERBPs and RAS was calculated, and the DERBP–RAS relationship pairs satisfying the absolute correlation coefficient value of ≥ 0.6 and p*-*value of ≤ 0.01 were screened.

### Functional enrichment analysis

KOBAS 2.0 server was used to perform functional enrichment analysis to identify the Gene Ontology (GO) terms and Kyoto Encyclopedia of Genes and Genomes (KEGG) pathways for screening the functional categories of DEGs [51]. The enrichment of each term was defined using the hypergeometric test and Benjamini–Hochberg FDR controlling procedure.

### Other statistical analysis

Principal component analysis (PCA) was performed using the R package factoextra (https://cloud.r-project.org/package=factoextra) to display the clustering of samples with the first two components. The reads of each gene in samples were normalized using tags per million. An in-house script (sogen) was used to visualize next-generation sequencing data and genomic annotations. Clustering based on Euclidean distance was performed using the pheatmap package in R (https://cran.r-project.org/web/packages/pheatmap/index.html). The student’s t-test was used to identify the divergence between OA and healthy samples.

### Validation of gene expression by RT-qPCR

Meniscus samples were collected from patients with OA who were undergoing total knee arthroplasty (TKA) and those with sports injuries who were undergoing arthroscopic partial meniscectomy as normal controls at Renmin Hospital of Wuhan University. The medical ethical committee of Renmin Hospital of Wuhan University reviewed and approved the protocol and design of this study. All patients provided written informed consent before surgery.

RT-qPCR was performed to validate target gene expression. A reverse transcription kit (R323–01, Vazyme, China) was used to synthesize cDNA. The thermocycler (T100, Bio-Rad, USA) conditions were as follows: 42˚C for 5 min, 37˚C for 15 min, and 85˚C for 5 s. Then, ABI QuantStudio 5 (ThermoFisher, USA) was used to perform qPCR. The cycling conditions were as follows: denaturing at 95˚C for 10 min and 40 cycles of denaturing at 95˚C for 15 s and annealing and extension at 60˚C for 1 min. Three technical replicates were used for each sample. The relative expression of each transcript was calculated using the 2^−ΔΔCT^ method. Glyceraldehyde-3-phosphate dehydrogenase was used as the internal reference gene for normalization [52]. Two-way analysis of variance or student’s t-test was used to perform statistical analysis with GraphPad Prism software (Version 8.0, San Diego, USA). All statistical significance was specified as *p* < 0.05.

Furthermore, RT-qPCR was performed to validate AS events. A boundary-spanning primer was used for the sequence encompassing the junction of constitutive and alternative exons, and an opposing primer in the constitutive exon was used to detect alternative isoforms. The boundary-spanning primer was designed based on the “model exon” to detect model splicing or based on the “altered exon” to detect altered splicing.

### Electronic supplementary material

Below is the link to the electronic supplementary material.

Supplementary Material 1

Supplementary Material 2

Supplementary Material 3

Supplementary Material 4

Supplementary Material 5

## Data Availability

The publicly available sequence data files of four human OA meniscus samples and four healthy meniscus samples from GSE185064 were downloaded from the Sequence Read Archive (SRA) (https://www.ncbi.nlm.nih.gov/geo/query/acc.cgi?acc=GSE185064). All data generated or analyzed during this study are included in this article and its Supplemental information files. Further inquiries for data are available from the corresponding author on reasonable request.

## Abbreviations

AS: Alternative splicing
OA: Osteoarthritis
RBP: RNA binding protein
DERBP: Differentially expressed RNA binding protein
DEG: Differential expressed genes
RAS: Regulatory alternative splicing
FPKM: Fragments per kilobase of exon per million mapped fragments
FDR: False discovery rate
FC: Fold change
SUVA: Splice sites usage variation analysis
pSAR: Proportion of each SUVA AS event reads
PCA: Principal component analysis
GO: Gene Ontology
KEGG: Kyoto Encyclopedia of Genes and Genomes
TKA: Total knee arthroplasty
