# Supplementary figures and images for: RNA-binding proteins potentially regulate the alternative splicing of apoptotic genes during knee osteoarthritis progression
Source: BMC Genomics. 2024 Mar 19;25:293. doi: 10.1186/s12864-024-10181-w (PMC10949708; doi:10.1186/s12864-024-10181-w)

A

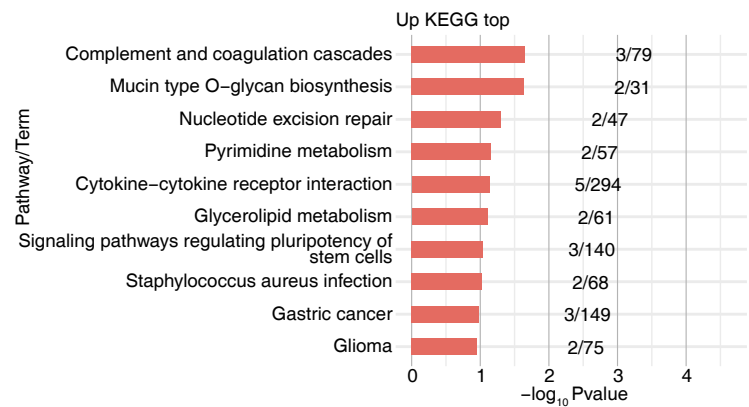

B

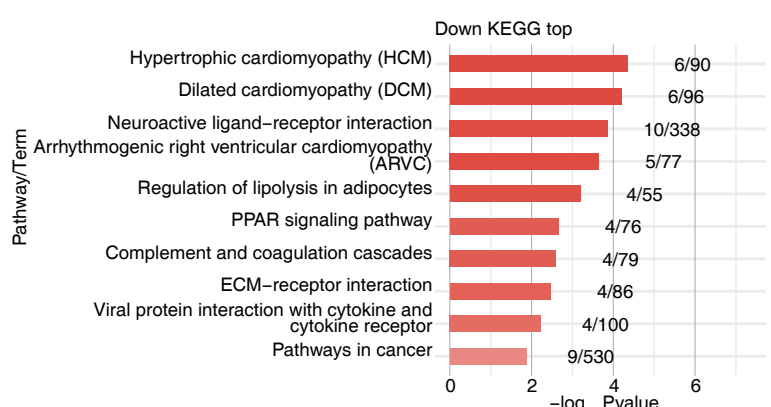

Supplement: Supplementary file 1 — Supplementary Material 1 [file 12864_2024_10181_MOESM1_ESM.pdf]

**A**

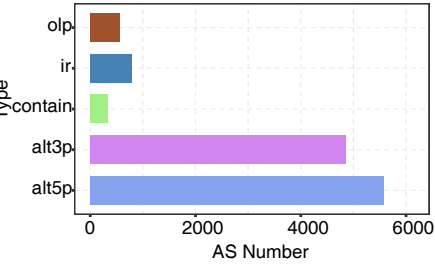

**B**

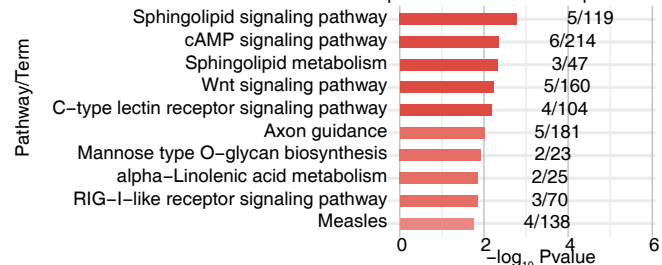

Supplement: Supplementary file 2 — Supplementary Material 2 [file 12864_2024_10181_MOESM2_ESM.pdf]

A

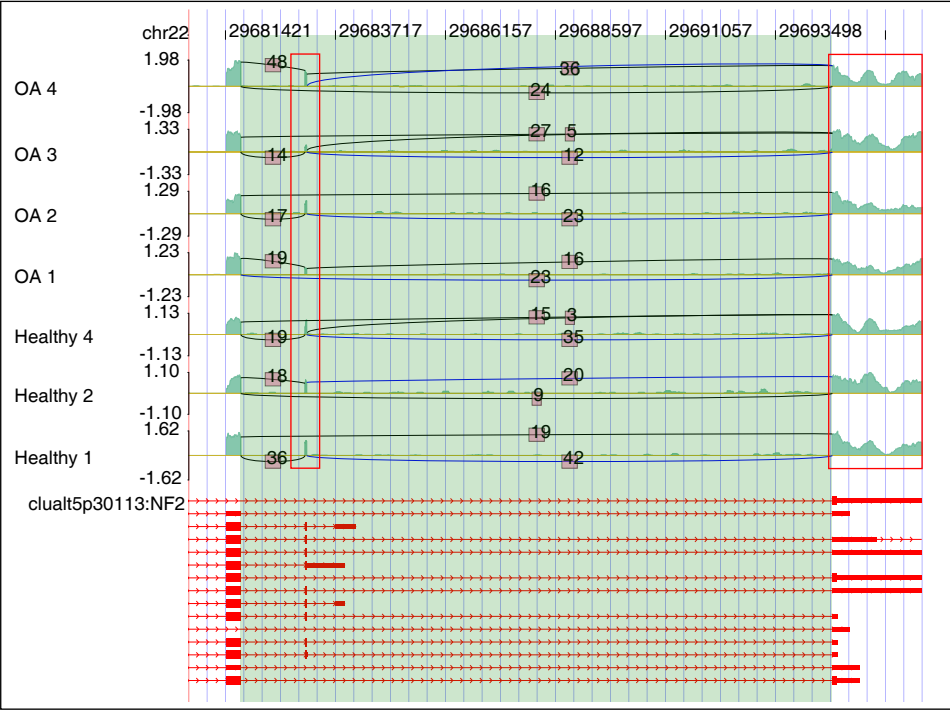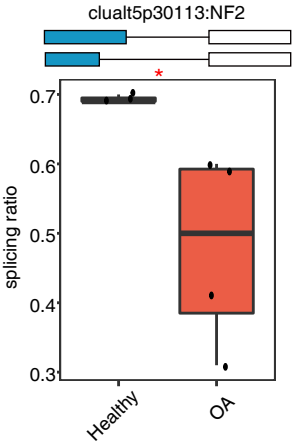

Supplement: Supplementary file 3 — Supplementary Material 3 [file 12864_2024_10181_MOESM3_ESM.pdf]

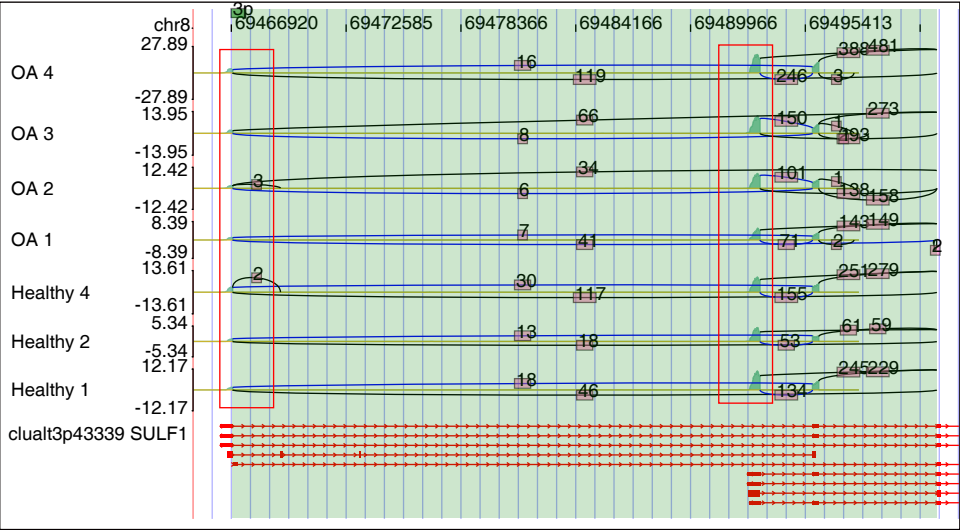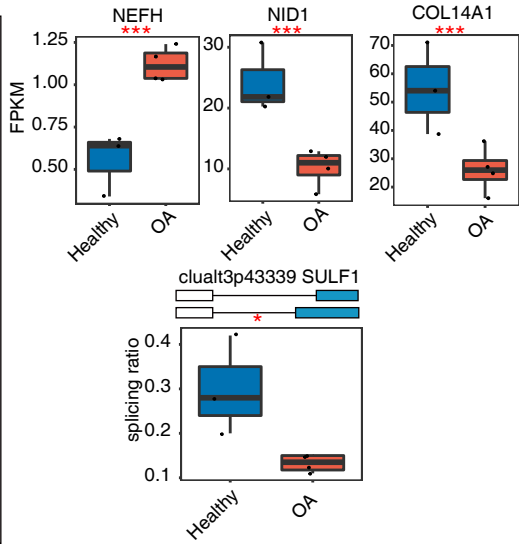

Supplement: Supplementary file 4 — Supplementary Material 4 [file 12864_2024_10181_MOESM4_ESM.pdf]
