# Supplementary material for: RNA-binding proteins potentially regulate the alternative splicing of apoptotic genes during knee osteoarthritis progression
Source: BMC Genomics. 2024 Mar 19;25:293. doi: 10.1186/s12864-024-10181-w (PMC10949708; doi:10.1186/s12864-024-10181-w)
Supplement: Supplementary file 5 — Supplementary Material 5 [file 12864_2024_10181_MOESM5_ESM.pdf]

Figure Legends

Supplemental Fig. S1 RNA sequencing of human meniscus cells in osteoarthritis (OA) and healthy samples.
(A) Bar plot showing the most enriched KEGG pathways of upregulated DEGs.
(B) Bar plot showing the most enriched KEGG pathways of downregulated DEGs.

Supplemental Fig. S2 Identification of predominant regulatory alternative splicing (RAS) events between OA and healthy samples.
(A) Bar plot showing the number of different RAS types identified by SUVA in healthy samples.
(B) Bar plot showing the most enriched KEGG pathways of the RAS with pSAR $\geq$ 50%.

Supplemental Fig. S3 Alternative splicing (AS) of apoptotic genes in osteoarthritis (OA).
The read distribution and splicing ratio of apoptotic gene NF2. The red box in the left panel indicates the splicing area of the difference; the boxplot in the right panel shows the splicing ratio. *: $p < 0.05$.

Supplemental Fig. S4 Covariation analysis of differentially expressed RNA binding protein (DERBP) and regulatory alternative splicing (RAS) in apoptotic pathway of OA.
The read distribution of the apoptotic RAS gene SULF1 is showing in the left panel. The red boxes indicate the splicing area of the difference. The boxplots in the right panel show the FPKM of the coexpressed RBP genes NEFH, NID1, and COL14A1 and the splicing ratio of SULF1. *: $p < 0.05$, **: $p < 0.01$, ***: $p < 0.001$.
